# Application of Stress Coping Ability as a Conduit between Goal Orientation and Play Effectiveness among Polish Soccer Players

**DOI:** 10.3390/ijerph19127341

**Published:** 2022-06-15

**Authors:** Paweł Kalinowski, Olga Bugaj, Łukasz Bojkowski, Yee Cheng Kueh, Garry Kuan

**Affiliations:** 1 Campus Hamburg, Macromedia University of Applied Sciences, 20095 Hamburg, Germany; pkalinowski@stud.macromedia.de; 2 Department of Athletics, Strength and Conditioning, Poznan University of Physical Education, 61-871 Poznan, Poland; bugaj@awf.poznan.pl; 3 Department of Psychology, Poznan University of Physical Education, 61-871 Poznan, Poland; bojkowski@awf.poznan.pl; 4 Biostatistics and Research Methodology Unit, School of Medical Sciences, Universiti Sains Malaysia, Kubang Kerian 16150, Malaysia; 5 Exercise and Sports Science Programme, School of Health Sciences, Universiti Sains Malaysia, Kubang Kerian 16150, Malaysia

**Keywords:** soccer, task-oriented, ego-oriented, psychology, stress coping

## Abstract

Background: Athletes should be distinguished by their capacity to deal with stress effectively. Motivated soccer players will employ stress-coping strategies that are linked to their ability to perform at a high level. The purpose of this study is to determine the relationship between a player’s specific goal orientation, coping in sports, and the effectiveness of play during competition. Methods: The study enrolled 122 male elite soccer players at the championship level who were between the ages of 16 and 19. All participants completed the Polish version of the Task and Ego Orientation in Sport Questionnaire, the Polish version of the Coping Inventory for Competitive Sport Questionnaire, the CISS questionnaire, and Szwarc’s observation sheet for evaluating soccer performance. Results: The results showed that the task-based stress-coping mode partially mediated the relationship between task motivation and the observed effectiveness of players in soccer. Conclusion: From an applied perspective, the data on the relationship between selected mental factors and soccer performance point to a possible direction of work for players aspiring to the highest level of performance.

## 1. Introduction

The motivation for achievements in sports has a special place. It is associated with setting increasingly higher goals for oneself and delaying external gratification. Athletes with a high level of motivation to achieve goals through ingenious and innovative solutions take on a sense of responsibility for their actions [1]. One of the most applicative theories of motivation is the Achievement Goal Theory [2], which proposes that the deliberate behaviour of an athlete during training and competition depends mainly on his or her preferences and goals. Nicholls [2] claims that motivation to achieve goals concerns behaviours that favour development. According to him, ego and task orientations can be distinguished [3]. Ego orientation puts emphasis on presenting high skills without considering involvement, whereas task orientation involves focusing on the personal development of skills [4,5]. Individuals with a strong ego orientation interpret failure as a lack of abilities or talent [4,5]. By contrast, task-oriented individuals are identified by their intrinsic motivation and enjoyment of their work. They are willing to make long-term commitments because the prospect of developing their competences excites them [6]. In light of studies, task orientation is more beneficial for soccer players [7], as those involved in the learning process, development of motor properties, and increasing technical and tactical skills have a better chance of further development in case of failure or defeat.

Task-oriented motivation is particularly significant during sports competitions when a difficult situation occurs. Studies have shown that people with a task-oriented approach, in spite of the difficulties they face in solving a problem, will continue the previously applied tactics and are more effective than those characterised by having a personal approach, who usually give up and report a willingness to switch activities [8,9]. The solutions applied by people with a task-oriented approach seem to be a more beneficial way of coping with stress in a difficult situation. During a game of soccer, a player constantly makes choices between more difficult or easier options for performing a task. Assuming that a player who is ego-oriented will aim to show their high skills and conceal their deficiencies when making their choice and performing the task, it can be stated that as difficulties appear, they will aim to switch the task for an easier one. On the other hand, a player who is task-oriented will accurately choose a task that is adequate for their capabilities, i.e., one in which the probability of success is the highest [7,10]. When a player with an ego-oriented approach chooses a difficult task, the consequences may lead to a low self-perception of capabilities and a huge disappointment, whereas a player with a task-oriented approach chooses a realistic task in the aspect of their own capabilities. Therefore, task orientation definitely works better in sports [4,11], although ego orientation may also be of high significance. In a Swedish study of soccer players, it was shown that a higher level of ego orientation favoured task-oriented strategies of coping with stress, whereas a lower level of ego favoured emotional strategies [12].

A review of the literature assumes that athletes should be characterised by an optimal level of task-oriented motivation and an optimal level of ego-oriented motivation [13]. Competitive athletes are characterised by their tenacity in pursuing their objectives and their dedication to training, regardless of the outcome goals [14]. It can be assumed that soccer players who are task-oriented will be more effective during the long-term process of reaching maximum capabilities. It should be noted that each player was analysed for both task orientation and ego orientation, which is aimed at comparing their own skills with those of other players, as well as competition and winning.

The presented statements suppose that a high level of task orientation will increase the possibility of finding better solutions in difficult situations during training and soccer matches. Appropriate task performance will, in turn, favour high effectiveness in fulfiling the tactical aims of a team. In the context of the presented premise, it can be characterised that the relationship between task orientation and the effectiveness of soccer players’ performance will be mediated by a task-oriented modus operandi of stress management. Thus, the optimal model of a player should incorporate highly oriented athletes who are characterised as those undertaking actions and focusing on a problem that ultimately favours an effective performance.

Recently, Isoard-Gautheur, Ginoux, and Trouilloud [15] conducted a study on peer motivational climate (peerMC) and sport-related well-being. They discovered that a task-driven peerMC is more likely to result in positive outcomes for athletes’ motivation and well-being, whereas an ego-driven climate is more likely to result in negative outcomes such as stress and burnout. Similar results were reported by Smith and Ullrich-French [16]. Therefore, coaches are encouraged to teach their athletes values such as cooperation, effort, mutual assistance, and social support [15,17].

In a previous study, it was discovered that stress management is partially regulated by the relationships between soccer players’ personalities and on-field performance [15,18]. This has shifted our focus to the search for additional psychological factors that act as motivators. In reference to the objectives of the study, the following research questions were formulated: (1) Do task-oriented styles and strategies act as mediators between task orientation and performance effectiveness? (2) Do the dimensions of task-oriented styles, studied using the coping with stress questionnaire in sports, mediate the relationship between task motivation and performance effectiveness? (3) Do the dimensions of task-oriented strategies, studied using the coping with stress questionnaire in sports, mediate the relationship between task motivation and performance effectiveness?

## 2. Materials and Methods

### 2.1. Study Design

The study was approved by the Poznan University of Medical Sciences Ethics Committee and was conducted in accordance with the International Declaration of Helsinki guidelines. The data collection was performed in Wronki, Chorzów, and Szczecin (Poland). All participants signed the informed consent form in agreement to participate in this study.

### 2.2. Participants

A total of 122 youth male soccer players between the ages of 16 and 19 years volunteered to participate in the study. All participants have at least three to five years of competitive performance at the National championships. They were medallists in Polish championships in the under-17 and under-19 age categories.

### 2.3. Measures

#### 2.3.1. Effectiveness of Performance

The effectiveness of performance (EFF) of young soccer players was determined after the pilot study was carried out during championship games through video recordings. Each of the players was recorded using a video camera for 540 min. Then, using Szwarc’s observation method, the qualitative performance during play was converted into quantitative data [19]. The matches were recorded during championship matches between the participating teams by the author himself. The matches were played on full-size football pitches. The teams consisted of 11 players. The actions taken by the players on the pitch were recorded during video analysis and transferred to the “Szwarc Observation Sheet”. The research tool had a reliability value of 97.07% and validity of 98.02% [19,20]. On this basis, 15 indicators of players’ effectiveness in attack and defence were determined. During the study, all necessary procedures for the application of audio–visual technology were observed. The analysis of effectiveness was performed during games at the highest youth level in Poland. The technical and tactical aspects of the games were evaluated. They included the following performance indicators/elements: (1) shots at goal, (2) possession of the ball from an opponent in one-on-one play and overtaking passes on target, and (3) winning one-on-one duels in attacks. In addition, ineffective actions such as losing possession of the ball in one-on-one duels, inaccurate passing, dribbling with the ball, and staying offside were evaluated [19].

#### 2.3.2. Task and Ego Orientation in Sport Questionnaire (TEOSQ)

To determine the level of motivation and to differentiate the types of goal orientation in athletes, the Task and Ego Orientation in Sport Questionnaire (TEOSQ) was used [21]. The translation of the Polish version was performed by Tomczak et al. [6] to distinguish two types of motivation in sports: task-oriented and ego-oriented. The psychometric properties of the Polish-TEOSQ were presented at the 13th FEPSAC Congress [22]. The Cronbach’s alpha was 0.81 (task orientation) and 0.84 (ego orientation), with an interval of two weeks. Using test–retest reliability resulted in ICC = 0.86 (task orientation) and ICC = 0.86 (ego orientation) [6].

#### 2.3.3. Coping Inventory for Competitive Sport Questionnaire (CICS)

The Coping Inventory for Competitive Sport is a questionnaire that assesses stress-coping styles in sport (CICS—styles) and strategies, i.e., actions taken during sports competitions (CICS—strategies) [23]. The article uses the CICS style and strategies questionnaire, which was created on the basis of the original CISS research tool [23]. The current study used the Polish version adapted by Knittel and Guszkowska [23]. The questionnaire contains 39 items and assesses 10 strategies and 10 styles [23,24]. The Coping Inventory for Competitive Sport (CICS) is a widely used questionnaire for assessing how athletes cope with stress. It has high validity and reliability in a variety of languages. The Cronbach’s alpha internal consistency coefficient values of the Polish version ranged between 0.63 and 0.89 [23].

### 2.4. Data Analysis

Mediational analysis was used to analyse the data, in which a significant mediational effect occurred when a statistically significant indirect effect was achieved. This is the multiplicative effect of the relationship between an independent variable and the mediator (the “a” path), and the relationship between the mediator and a dependent variable under an independent variable (the “b” path indirect effect: a × b). The percentile bootstrap was used to determine the significance of the indirect effect, and to construct a confidence interval [25]. The indirect effect is statistically significant at the level of 0.05 if the 95% confidence interval does not contain zero. The confidence interval was determined in an analysis using 5000 bootstrap samples with the application of Andrew Hayes’s Macra process [25].

In addition, the study compared the mediational effects obtained in single mediations using path analysis and structural equations modelling (SEM). The fit of models was evaluated using the Chi-square test, CFI (Comparative Fit of Index), and RMSEA (Root Mean Square Error of Approximation). The Chi-square test was insignificant, the CFI was greater than 0.90, and the RMSEA was less than 0.08, indicating that the model was adequately fitted to the data [26]. Then, the insignificant paths were omitted from the model. Finally, the path analysis was analysed using AMOS version 23.0. Then, the models were used to summarise the obtained effects.

## 3. Results

Table 1 summarises the mediational analysis of the relationship between task orientation, styles of coping with stress, and effectiveness of performance among soccer players.

In the descriptive analysis, significant relationships were noted between task orientation and effectiveness of performance (β = 0.20; *p* < 0.05). In the relationship between task orientation and the styles of coping with stress that is focused on a task, from an overall perspective, a statistically significant indirect effect was observed for the task-oriented style (0.0732; 95% CI (0.0153; 0.1568)). Task-oriented motivation was positively correlated with the task-oriented style from an overall perspective of CISS (β = 0.28; *p* < 0.001), which is related to effectiveness of performance (β = 0.26; *p* < 0.01) (see Table 1). For thought control, a significant indirect effect was observed (0.1113; 95% CI (0.0291; 0.2118)). Motivation toward task orientation was positively associated with thought control (β = 0.39; *p* < 0.001), which was positively correlated with the effectiveness of performance (β = 0.28; *p* < 0.01) (see Table 1). For logical analysis, a significant indirect effect was observed (0.0805; 95% CI (0.0140; 0.1758)). Motivation towards task orientation was positively associated with improvement in logical analysis (β = 0.34; *p* < 0.001). Logical analysis was also positively associated with effectiveness of performance (β = 0.23; *p* < 0.05) (Table 1). Other relationships between task-oriented motivation, styles focused on a task, and the effectiveness of performance, and intervening variables such as effort expenditure, mental imagery, seeking support, and relaxation were found to have insignificant mediating effects (see Table 1).

Table 2 presents a mediational analysis of the relationship between goal orientation, strategies of coping with stress, and the effectiveness of performance among soccer players.

A significant intermediate effect was observed between goal orientation and task-based labour input strategy and operational efficiency. Motivation toward task orientation was positively associated with effort expenditure (β = 0.34; *p* < 0.001). Effort expenditure was also associated with effectiveness of performance (β = 0.28; *p* < 0.01) (see Table 2). For thought control, a significant indirect effect was observed (0.0630; 95% CI (0.0025; 0.1578)). Motivation towards task orientation was positively associated with thought control (β = 0.28; *p* < 0.01), which was positively associated with effectiveness of performance (β = 0.22; *p* < 0.05) (see Table 2). However, intervening variables such as mental imagery, seeking support, relaxation, and logical analysis showed insignificant mediating effects (see Table 2).

**Figure 1:**
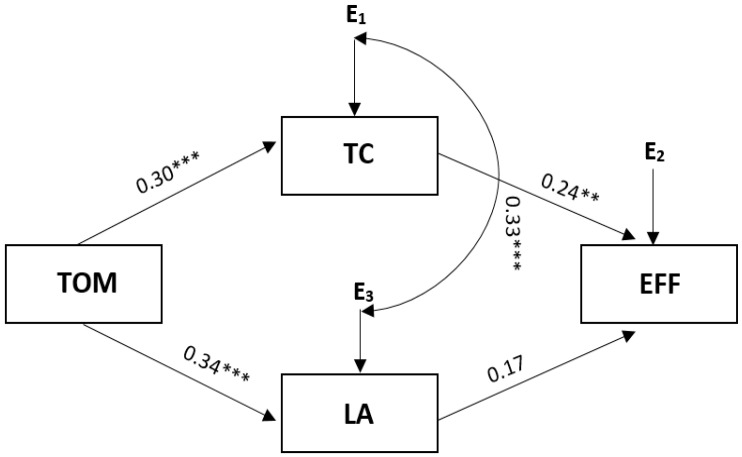
The summarising model for task orientation and styles of coping with stress. Note. Chi^2^ = 0.286; df = 1; *p* = 0.593; CFI model = 1.00; RMSEA = 0.001; indirect effect for task motivation = 0.16; 95% CI [0.067; 0.247]. Coefficients: effectiveness R^2^ = 0.12; TC R^2^ = 0.16; LA R^2^ = 0.12; LA—logical analysis; TC—thought control; EFF—effectiveness of performance; TOM—task-oriented motivation. ** *p* < 0.01; *** *p* < 0.001.

The path model below summarises the mediating effects (Figure 1). This model is presented to summarise the relationships between task orientation, coping with stress, and the effectiveness of performance among soccer players.

The presented model demonstrated excellent fit with the data: Chi^2^ = 0.286; df = 1; *p* = 0.593; RMSEA = 0.001; and CFI = 1.00. The variables accounted for 12% of the effectiveness of performance among soccer players (see Figure 1). Additionally, as demonstrated by the analysis of modification indices, the correlation between errors for mediators is permitted.

As with single mediations, task motivation was found to be positively associated with logical analysis and thought control, increasing the chance of high effectiveness. Additionally, a direct correlation was observed between task-oriented motivation and the effectiveness of performance among soccer players (similar to the single mediational models).

**Figure 2:**
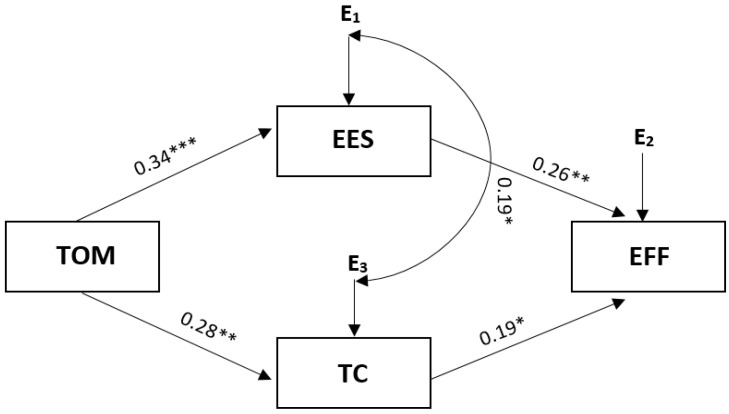
The summarising model of task orientation for strategy of coping with stress. Note. Chi^2^ = 0.465; df = 1; *p* = 0.495; CFI model = 1.00; RMSEA = 0.00; indirect effect for task-oriented motivation = 0.143; 95% CI [0.061;0.247]. Coefficients: effectiveness R^2^ = 0.13; EES R^2^ = 0.12; TCS R^2^ = 0.08; EES—effort expenditure strategies; TC—thought control; EFF—effectiveness of performance; TOM—task-oriented motivation. * *p* < 0.05; ** *p* < 0.01; *** *p* < 0.001.

Figure 2 below shows a summary model of strategies for coping with stress. The presented model demonstrated excellent fit with the data: Chi^2^ = 0.465; df = 1; *p* = 0.495; RMSEA = 0.001; and CFI = 1.00. The variables explained 13% of the effectiveness of performance among soccer players. As with single mediations, task-oriented motivation increased effort expenditure and thought control and increased the likelihood of high effectiveness (see Figure 2). There was a direct positive relationship between task-oriented motivation and the effectiveness of performance among soccer players.

## 4. Discussion

The primary objective of the study was to examine the relationship between specific goal orientation and the effectiveness of performance, both of which are associated with ways of coping with stress, as well as to provide answers to detailed research questions.

Coping with stress and motivation are factors that determine success in soccer, where stressful experiences often lead to negative consequences in everyday life and eudaimonic and hedonic well-being [27]. However, there are few scientific studies that examine the relationship between task-oriented motivation and the effectiveness of performance.

There was a significant correlation between task-oriented motivation and the effectiveness of performance. The results from the current study showed that the higher the level of task-oriented motivation, the higher the effectiveness of performance. These findings corroborate earlier findings by Diener and Dweck [8,9].

The statistical analysis in this study supported only a portion of the hypothesis concerning our first research question, “Do task-oriented styles and strategies act as mediators between task orientation and performance effectiveness?” The relationship between task-oriented motivation and effectiveness was explained in the survey using the CISS questionnaire for task-oriented styles. It was demonstrated that soccer players with higher task-oriented motivation demonstrated, to a larger extent, task-oriented ways that lead to higher effectiveness of performance. These findings also corroborate Aldwin’s [28] and Knittel and Guszkowska’s [23,24] early reports. They do not, however, corroborate the results of the study on Swedish soccer players, in which a higher level of ego was associated with a preference for task-oriented stress management strategies [12]. At the same time, it is worth noting that soccer players showing task-oriented motivation have higher emotional intelligence and lower levels of anxiety related to sports performance [29].

The hypothesis included in the second research question, “Do the dimensions of task-oriented styles, studied using the coping with stress questionnaire in sports, mediate the relationship between task motivation and performance effectiveness?” was partially confirmed. Thought control and logical analysis explained the relationship between task-oriented motivation and the effectiveness of performance. It was not confirmed, however, for other dimensions of the task-oriented style, such as seeking support, relaxation, mental imagery, and effort expenditure. The results of this study partially confirmed the reports by Albuquerque et al. [13].

The hypothesis included in research question No. 3, “Do the dimensions of task-oriented strategies, studied using the coping with stress questionnaire in sports, mediate the relationship between task motivation and performance effectiveness?” was confirmed only in cases of task-oriented strategies, such as effort expenditure and thought control, while it was not confirmed for the following strategies: mental imagery, seeking support, relaxation, and logical analysis. The explanation was sought in these relationships and the characteristics of the sport. Technical and tactical actions of players during games take place in varying situations, which depend on many factors. Sports competitions last 90 min, or 120 min with extra time, and players need to maintain full concentration and solve problematic situations throughout the whole game. Irrespective of the results and the opposition level, the players should be motivated all the time. The use of task-oriented strategies, which in these conditions can have good results, seems highly justified. Thus, it may be assumed that a high level of task orientation gets players closer to the achievement of their objectives. This was confirmed in Roy’s [30] and Aldwin’s [28] reports. In the current study, task-oriented motivation was found to be significantly associated with performance effectiveness. Thus, it may be assumed that a high level of task-oriented motivation favours high effectiveness in soccer. However, sports outcome is better explained by variables other than task-oriented motivation.

In terms of application, the information concerned the relationship between selected mental factors (motivation, styles, and strategies of coping with stress) and the effectiveness of performance in sports [31,32]. The results of this study (the relationship between motivation and the effectiveness of performance) may aid in the development of players and the coaching staff’s ability to conduct an effective training process. Moreover, from the applicative point of view, they may complement the coaching repertoire in terms of the relationship between motivation, effort expenditure, and effectiveness of performance concerning work with the players and the team. The final practical recommendation is to emphasise task-oriented behaviours in soccer, which are most significant for the effectiveness of performance in the soccer game.

This study’s limitation may be the absence of cross-sectional studies and the study population’s high level of sports. As a result, it is recommended that in future studies, researchers expand the number of mental properties studied (including grit and self-efficacy) and the number of participants representing different sporting levels, including both sexes. Another limitation is that the sample size of the present study (i.e., *n* = 122) is not above the median sample size other researchers have used for SEM analysis (i.e., about 200 cases) [33]. This was due to the difficulty of recruiting large numbers of eligible youth male soccer players during the data collection period. However, based on the SEM analysis, we found that the model fit the data well. In future studies, when more study variables are included in the hypothesised SEM model, which will increase the complexity of the model, a larger sample size will be required for a stable model.

## 5. Conclusions

In light of the results and analyses presented above, we can conclude that the present study established a direct relationship between task-oriented motivation and the effectiveness of performance among young soccer players. In addition, task-oriented approaches (an overall task-oriented style, thought control, effort expenditure, and logical analysis) mediated the relationship between task-oriented motivation and player effectiveness. Thus, when compared to players with a lower level of task-oriented motivation, those with a higher level of task-oriented motivation frequently coped well with stress through task-oriented approaches. On the other hand, soccer players who frequently exhibited elements of the task-oriented style demonstrated a higher level of performance effectiveness.

## Figures and Tables

**Table 1:** Mediational analysis of the relationship between task orientation, styles of coping with stress, and performance effectiveness.

Dependent Variable	Independent Variable	Intervening Variable	a	b	c’	c	Indirect Effect (a × b) 95% CI	F/R^2^
EFF	Task-oriented motivation	Mental imagery	0.22 *	0.17	0.16	0.20 *	0.038 [−0.002; 0.105]	4.19 */0.06
		Effort expenditure	0.34 ***	0.28 **	0.10	0.20 *	0.095 [0.031; 0.175]	7.15 **/0.11
		Thought control	0.28 **	0.22 *	0.13	0.20 *	0.063 [0.001; 0.160]	5.48 **/0.08
		Seeking support	0.18 *	0.07	0.18 *	0.20 *	0.013 [−0.020; 0.061]	2.74/0.04
		Relaxation	0.39 ***	0.01	0.19	0.20 *	0.005 [−0.064; 0.081]	2.39/0.04
		Logical analysis	0.24 **	0.18	0.15	0.20 *	0.044 [−0.003; 0.118]	4.35 */0.07

*p < 0.05; ** *p* < 0.01; *** *p* < 0.001; a—relationship between independent variable and mediator, b—relationship between mediator and dependent variable with statistical control for independent variable, c’—relationship between independent and dependent variable with statistical control for mediator, c—relationship between independent variable and dependent variable, a × b—indirect effect, F/R^2^—value of F-test statistics for the regression model with independent variable and mediation (determination coefficient), EFF—effectiveness of performance.

**Table 2:** Mediational analysis for the relationship between task orientation, strategies of coping with stress, and effectiveness of performance.

Dependent Variable	Independent Variable	Intervening Variable	a	b	c’	c	Indirect Effect (a × b) 95% CI	F/R^2^
EFF	Task-oriented motivation	Task-oriented style	0.28 ***	0.26 **	0.12	0.20 *	0.073 [0.014; 0.161]	6.53 **/0.10
		Mental imagery	0.19 *	0.19 *	0.16	0.20 *	0.036 [−0.006; 0.1006]	4.70 */0.07
		Effort expenditure	0.37 ***	0.15	0.14	0.20 *	0.056 [−0.013; 0.135]	3.72 */0.06
		Thought control	0.39 ***	0.28 **	0.08	0.20 *	0.111 [0.029; 0.209]	7.05 **/0.10
		Seeking support	0.20 *	0.12	0.17	0.20 *	0.024 [−0.017; 0.079]	3.27 */0.05
		Relaxation	0.31 ***	0.09	0.17	0.20 *	0.028 [−0.027; 0.101]	2.85/0.04
		Logical analysis	0.34 ***	0.23 *	0.11	0.20 *	0.081 [0.014; 0.179]	5.65 **/0.09

*p < 0.05, ** *p* < 0.01, *** *p* < 0.001; a—relationship between independent variable and mediator, b—relationship between mediator and dependent variable with statistical control for independent variable, c’—relationship between independent variable and dependent variable with statistical control for mediator, c—relationship between independent variable and dependent variable, a × b—indirect effect, F/R^2^—value of F test statistics for the regression model considering independent variable and mediation (determination coefficient), EFF—effectiveness of performance.

## Data Availability

The study data are available upon request from the authors.
